# Odontogenic Cervicofacial Necrotizing Fasciitis: Microbiological Characterization and Management of Four Clinical Cases

**DOI:** 10.3390/pathogens11010078

**Published:** 2022-01-09

**Authors:** Sebastian Böttger, Silke Zechel-Gran, Daniel Schmermund, Philipp Streckbein, Jan-Falco Wilbrand, Michael Knitschke, Jörn Pons-Kühnemann, Torsten Hain, Markus Weigel, Can Imirzalioglu, Hans-Peter Howaldt, Eugen Domann, Sameh Attia

**Affiliations:** 1 Department of Oral and Maxillofacial Surgery, Justus-Liebig-University Giessen, University Hospital Giessen, Klinikstrasse 33, 35392 Giessen, Germany; Daiel.Schmermund@uniklinikum-giessen.de (D.S.); Philipp.Streckbein@uniklinikum-giessen.de (P.S.); Jan-Falco.Wilbrand@uniklinikum-giessen.de (J.-F.W.); Michael.Knitschke@uniklinikum-giessen.de (M.K.); HP.Howaldt@uniklinikum-giessen.de (H.-P.H.); sameh.attia@dentist.med.uni-giessen.de (S.A.); 2 Institute of Medical Microbiology, Justus-Liebig-University Giessen, 35392 Giessen, Germany; Silke.Zechel@mikrobio.med.uni-giessen.de (S.Z.-G.); Torsten.Hain@mikrobio.med.uni-giessen.de (T.H.); Markus.Weigel@mikrobio.med.uni-giessen.de (M.W.); Can.Imirzalioglu@mikrobio.med.uni-giessen.de (C.I.); 3 Institute of Medical Informatics, Justus-Liebig-University Giessen, 35392 Giessen, Germany; Joern.Pons@informatik.med.uni-giessen.de; 4 German Center for Infection Research (DZIF), Justus-Liebig-University Giessen, 35392 Giessen, Germany; Eugen.Domann@mikrobio.med.uni-giessen.de; 5 Institute of Hygiene and Environmental Medicine, Justus-Liebig-University Giessen, Schubertstrasse 81, 35392 Giessen, Germany

**Keywords:** fasciitis, necrosis, abscess, cellulitis, microbiome, 16S-rRNA gene analysis

## Abstract

Necrotizing fasciitis of the head and neck is a rare, very severe disease, which, in most cases, originates from odontogenic infections and frequently ends with the death of the patient. Rapid surgical intervention in combination with a preferably pathogen-specific antibiotic therapy can ensure patients’ survival. The question arises concerning which pathogens are causative for the necrotizing course of odontogenic inflammations. Experimental 16S-rRNA gene analysis with next-generation sequencing and bioinformatics was used to identify the microbiome of patients treated with an odontogenic necrotizing infection and compared to the result of the routine culture. Three of four patients survived the severe infection, and one patient died due to septic multiorgan failure. Microbiome determination revealed findings comparable to typical odontogenic abscesses. A specific pathogen which could be causative for the necrotizing course could not be identified. Early diagnosis and rapid surgical intervention and a preferably pathogen-specific antibiotic therapy, also covering the anaerobic spectrum of odontogenic infections, are the treatments of choice. The 16S-rRNA gene analysis detected significantly more bacteria than conventional methods; therefore, molecular methods should become a part of routine diagnostics in medical microbiology.

## 1. Introduction

Necrotizing fasciitis is a severe, life-threatening disease that can lead to a highly septic clinical presentation with extensive tissue necrosis in various areas of the body, and a poor outcome [1]. It is defined as a bacterial infection of the superficial fascial layer and the adjacent cutaneous tissues leading to fulminant, devastating and rapidly progressive necrosis of the affected tissues [2,3]. The disease is most common in the trunk, the extremities and the perineum [2,4], while it is rare in the head and neck region, accounting for only 1–10% of cases [5]. The majority of cases of cervicofacial necrotizing fasciitis are caused by an odontogenic focus [6,7,8]. Thus, similar to other odontogenic infections, the diagnosis is usually based on clinical and radiological findings [7], but the disease can easily be misdiagnosed as a common odontogenic abscess, cellulitis or erysipelas in its early stages [9]; thus, the resulting danger for the patient might be underestimated. Only advanced stages show the almost pathognomonic picture of necrotizing fasciitis with small purple spots [2], dark hemorrhagic blisters, crepitus, complete anesthesia and dusky necrosis of the affected skin [9,10,11]. Concomitant septic symptoms such as hypotension, tachypnea and impaired consciousness usually already indicate a life-threatening condition [7]. Numerous authors, therefore, have pointed out the importance of rapid diagnosis and prompt intervention [2,7,10], since a delay in therapy usually leads to a significant decrease in the probability of survival [12]. The cornerstones of therapy of odontogenic necrotizing fasciitis are consequent surgical exposure of the affected fascia with radical removal of necrotic tissue and an empirical broad-spectrum antibiotic therapy, which subsequently can be adjusted by targeted antibiotic treatment according to an antibiogram [7,11]. To be able to select an appropriate antibiotic, the microbial nature of the infection is of interest. Using cultural analyses, many authors have observed that odontogenic cervicofacial necrotizing fasciitis, similar to odontogenic abscesses, is caused polymicrobially by aerobic and anaerobic bacteria of the oral cavity [13]. Usually, two to three combined aerobic and anaerobic bacteria are identifiable [8], leading to a typical microbiological report consisting of alpha-hemolytic streptococci and obligate anaerobes such as *Prevotella* spp. and *Bacteroides* spp. [6,8]. Sometimes, culture is not even able to detect any bacteria at all [7,8]. Using more recent molecular biological methods, it has been possible to detect a much larger number of bacteria in the root canals of non-vital teeth [14] and in odontogenic abscesses [15,16] than was previously possible using only cultural methods alone [17,18].

Therefore, the purpose of this study was to microbiologically characterize four cases of extensive odontogenic necrotizing fasciitis using advanced molecular techniques in addition to standardized cultural analysis. Furthermore, we aim to describe how this rare but life-threatening disease can be diagnosed early and treated appropriately.

## 2. Results

In the period from April 2009 to December 2020, four extensive odontogenic infections with a severe course of disease and extensive formation of skin necrosis were treated in the Clinic for Oral and Maxillofacial Surgery of the university hospital. Two women and two men were hospitalized, aged between 38 and 74 years. Patient No. 2 was transferred intubated and ventilated from a peripheral hospital to the university hospital, while all other patients were primarily admitted to the university hospital. Patient No. 3 was transported with a severely reduced general condition to the clinic by ambulance service, while the remaining two patients presented themselves at the university hospital.

### 2.1. Clinical Findings

For all patients, an odontogenic cause of the inflammation could already be determined by the medical history and the routine clinical examination. One patient developed the inflammation due to a tooth extraction, based on the inflamed dental alveolus. Two patients had a decayed molar with chronic apical periodontitis, and one patient had an apical periodontitis originating from crowned lower incisors as the causative dental focus. All odontogenic foci were well documented clinically and radiologically (Table 1).

On admission, all patients already showed a considerable impairment of their general condition with extensive swelling and erythema (Table 1) as well as severe pain and tenderness in the area of the neck and massive dysphagia. Further, all patients showed a significant increase in inflammatory parameters and the LRINEC score (Table 1).

Due to the severe course of the disease, a computed tomography scan with a contrast medium was performed for all patients during the early course. Here, all patients showed contrast medium enhancement in the area of inflammation as well as fluid and gas inclusions along the fascial planes (Table 1). Tissue samples later confirmed the suspected diagnosis of necrotizing inflammation (Table 1).

### 2.2. Surgical Treatment

On admission, the patients presented themselves with varying degrees of disease progression. Two patients (patient Nos. 1 and 4) were initially treated with the diagnosis of an odontogenic abscess. The remaining two patients (Nos. 2 and 3) presented a complete picture of necrotizing fasciitis. These two patients underwent immediate surgery as an emergency, while the other two patients underwent surgical therapy as soon as possible, but not later than the first day after admission. In the case of the diagnosis “odontogenic abscess”, incisions and drains were performed using multiple Easy Flow drains. If necrotizing fasciitis was diagnosed, extensive deep incisions were performed, and fascial planes of the deep cervical fascia were exposed right down to the chest. In patient No. 2, multiple Easy Flow drains were also initially inserted, while in patient No. 3, all necrotically softened tissue was immediately removed radically. Furthermore, as recommended by numerous authors [7], a second-look procedure was performed on the first postoperative day. At this point, even in patient No. 2, the entire necrotic tissue was radically removed, exposing large parts of the pectoral musculature. Wound management was performed using Coldex^®^ hydro foam (Monomed NV, Hamont-Achel, Belgium), and later, coverage was realized using meshed split-thickness skin grafts. In patient No. 3, wound management was performed using negative wound pressure therapy (vacuum pump: V.A.C. ULTA^®^, 3M KCI, St. Paul, MN, USA), and later, wound coverage was also carried out using meshed split-thickness skin grafts.

The two cases initially diagnosed as an abscess showed their necrotizing potential only in the further course of the disease. Despite aggressive incision and drainage, patient No. 1 showed progressive necrotizing fasciitis after a few days, which required complete removal of the necroses and coverage with meshed split-thickness skin grafts in the further course. Patient No. 4 already showed massive redness of the skin and small black blisters over the punctum maximum of the inflammation at the time of the first surgical intervention. Despite aggressive incision and drainage with multiple Easy Flow drains, skin necroses several centimeters in size developed in the further course of the disease, which, however, remained locally confined to the neck. The necroses were locally resected, and the wounds were left to secondary wound healing.

### 2.3. Microbiological Findings and Ancillary Medical Treatment

**Figure 1:**
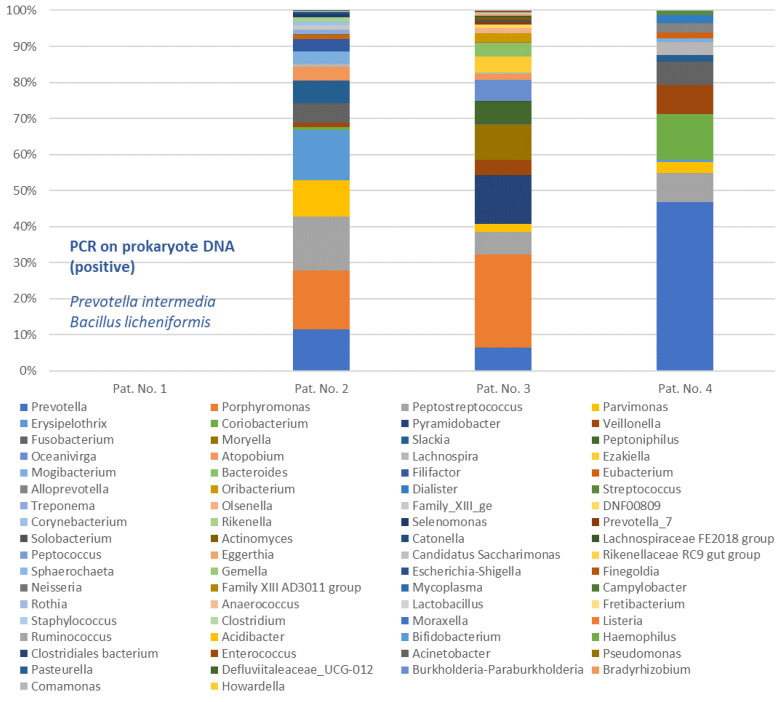
Microbiome of necrotizing fasciitis of three patients treated since 2016. PCR on prokaryote DNA identified *Prevotella intermedia* and *Bacillus licheniformis* in patient No. 1. The order of the bacterial genera in the legend corresponds to the mean of the relative abundances. Exact data are available in Table S1 of the Supplementary Materials.

Swabs were taken from all patients and sent to the microbiology laboratory for examination by culture. Table 1 and Table S1 show that all patients had a polymicrobial infection. With *Prevotella intermedia*, *Fusobacterium nucleatum* and alpha-hemolytic streptococci, typical aerobic and anaerobic bacteria of the oral microbiome, which were frequently associated with odontogenic infections [15,16,19], were detected in three patients. By contrast, patient No. 3 showed a rather atypical bacterial composition for an odontogenic infection with *Bacteroides thetaiotaomicron*, *Actinomyces turicensis* and *Staphylococcus epidermidis*. In addition to culture, PCR for prokaryote DNA was performed for the first patient, who had already been treated in 2009. In addition to the culture result, this allowed *Bacillus licheniformis* to be identified. The pus microbiome was determined for the three patients who were treated since 2016 using 16S-rRNA gene analysis. Figure 1 shows the microbiomes in the form of bar charts, and Table S1 shows the relative abundances of the reads of all bacterial genera. This indicates that, compared to other studies [17], many more bacteria could be detected than by culture alone. In these cases, *Prevotella*, *Porphyromonas*, *Fusobacterium*, *Parvimonas*, *Peptostreptococcus* and *Veillonella* appeared as typical pathogens of odontogenic infections [15]. Patient No. 2 also showed an abundance of *Erysipelothrix* and *Slackia*, while Patient No. 3 showed an abundance of *Pyramidobacter*, *Moryella* and *Peptoniphilus*. The genera *Bacteroides* and *Actinomyces,* which were recovered in culture, were also detected with a relative frequency of 3.9% and 0.5%, respectively. In patient No. 4, *Coriobacterium* was also predominant. Overall, our patients showed only negligible antibiotic resistance (Table 1). Susceptibility testing showed no resistance against antibiotics commonly used for odontogenic infections (ampicillin/sulbactam, clindamycin, metronidazole) [20] and against broad-spectrum antibiotics. All patients underwent empirical antibiotic therapy until susceptibility tests were performed. The initial diagnosis of abscess in patient Nos. 1 and 4 was initially treated with ampicillin and sulbactam according to the German guidelines for odontogenic infections [20]. Due to the exceptional severity of the disease, patient No. 4 was treated with an additional administration of metronidazole. In the case of the initial diagnosis of necrotizing fasciitis, clindamycin was administered in addition to a broad-spectrum antibiotic (patient No. 2: imipenem; patient No. 3: meropenem) to inhibit the protein biosynthesis of the bacteria [21] and thus to reduce the production of harmful enzymes. Furthermore, patient No. 2 was additionally treated with vancomycin due to a *Clostridium difficile*-associated colitis. In the susceptibility tests, all the substances used demonstrated a good efficacy against the bacteria, which were isolated by culture during the course.

### 2.4. Outcome

**Figure 2:**
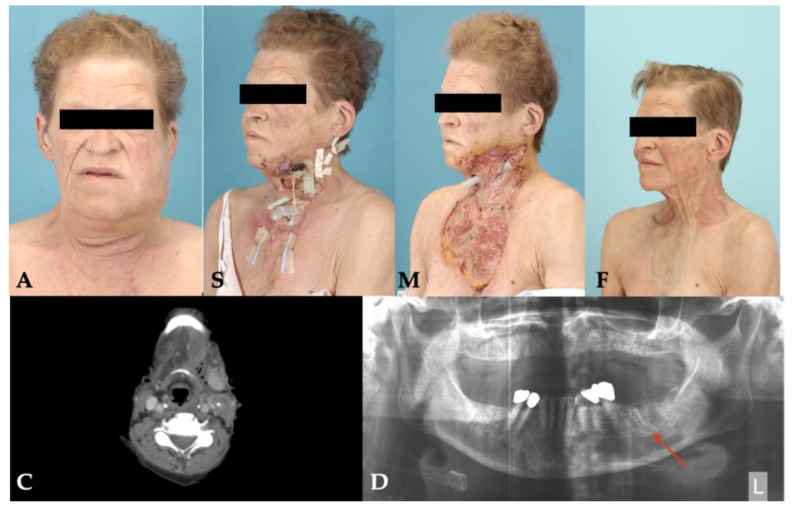
Clinical course and radiographs of patient No. 1: **A**: admission; **S**: surgery; **M**: maximal extent; **F**: final result; **C**: CT scan; **D**: dental focus (empty alveolus).

**Figure 3:**
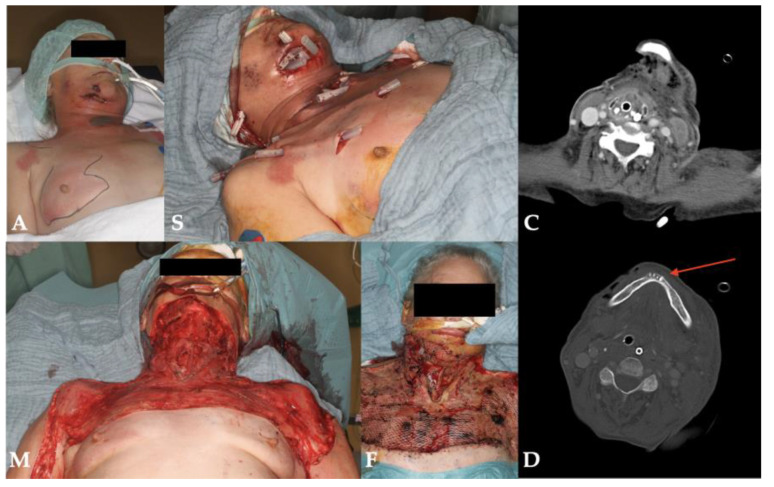
Clinical course and radiographs of patient No. 2: **A**: admission; **S**: surgery; **M**: maximal extent; **F**: final result; **C**: CT scan; **D**: dental focus (lower incisors).

**Figure 4:**
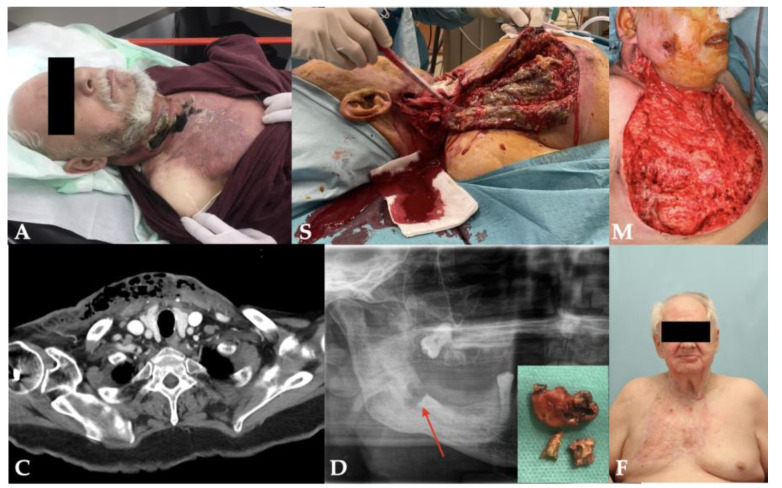
Clinical course and radiographs of patient No. 3: **A**: admission; **S**: surgery; **M**: maximal extent; **F**: final result; **C**: CT scan; **D**: dental focus (empty alveolus and decayed tooth 48).

**Figure 5:**
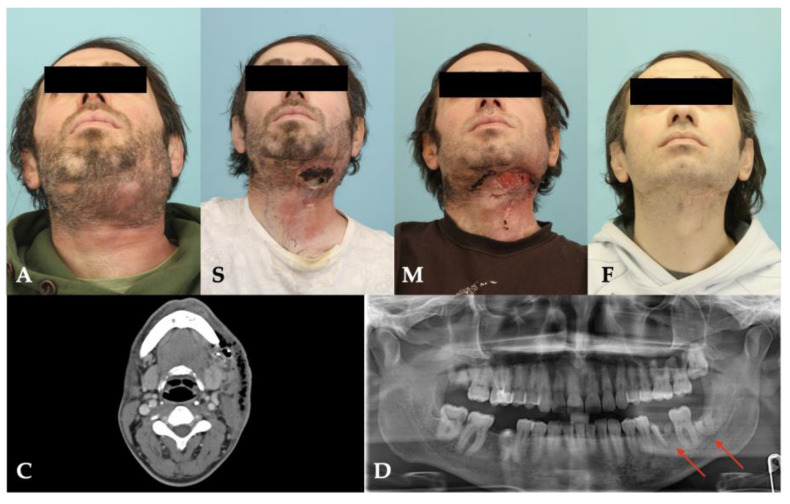
Clinical course and radiographs of patient No. 4: **A**: admission; **S**: surgery; **M**: maximal extent; **F**: final result; **C**: CT scan; **D**: dental focus (tooth remnants 36 and 38).

Three of four patients survived the necrotizing odontogenic infection, and one patient ultimately died due to septic multiorgan failure. The length of stay in the hospital ranged from 7 to 36 days which included intensive care of 1 to 21 days (Table 1). The surviving patients could be followed up for at least 15 months and showed scarring in the area of the former inflammations during the course, but functional deficits were minimal (Table 1). Patient No. 2 showed a drastically reduced general condition with extended necrotizing fasciitis already on admission. In addition to renal failure, she developed bilateral diffuse pneumonia, enteric ischemia and fulminant liver failure, leading to her final death as a result of septic shock and multiorgan failure. Figure 2, Figure 3, Figure 4 and Figure 5 show the entire clinical course of the four patients as well as representative CT images of the soft tissues and radiographs of the dental focus.

## 3. Discussion

### 3.1. Clinical Course of Necrotizing Fasciitis

Odontogenic infections are the most common infections in the head and neck region [22,23]. Most infections can be managed well on an outpatient basis, but patients with extensive abscesses require hospitalization [20,24]. The overall prognosis of these diseases is favorable if adequate surgical therapy and additional antibiotics are used [23]. Very rarely, instead of forming an abscess, odontogenic infections can lead to necrotizing fasciitis, which is described as a dreaded complication by many authors [2,7,10,22,23]. This results in acute life-threatening conditions, which can have severe consequences for the patient even in the case of survival [25]. In contrast to the common odontogenic abscess, the inflammation does not remain localized. The infection spreads through the tissue and extends to the level of the fasciae [12]. Destructive enzymes such as hyaluronidase and lipase allow the inflammation to spread rapidly at the level of the fasciae [12,26]. This results in septic thrombosis of the blood vessels of the skin crossing the fasciae, causing ischemia and necrosis of the skin [12,27]. Without appropriate therapy, the necrosis rapidly expands, leading to a highly septic clinical presentation and finally to the death of the patient [28]. Although healthy patients without pre-existing disorders can also develop the disease, older patients with pre-existing conditions are usually affected [2]. Diabetes mellitus followed by alcohol abuse with impaired liver function are reported as the most frequent predisposing disorders [8]. In addition, atherosclerosis, obesity, malnutrition, metastatic neoplasms, chronic renal failure and polymyositis are also mentioned [2]. Initially, only blurred skin redness and swelling appear, with smooth, tense and sometimes shiny skin [2]. Patients usually report disproportionately severe pain, typically extending widely beyond the edge of the skin redness, which provides an excellent clue to the true extent of disease [10]. In the further course, indurations, fluctuations and clear blister formations can appear [10]. If the disease progresses further, the almost pathognomonic picture of necrotizing fasciitis with small purple spots [2], dark hemorrhagic blisters, complete anesthesia of the affected skin, crepitus and dusky skin necrosis appears [9,10,11]. If left untreated, necrosis progresses to frank gangrene [10], and most patients develop sepsis with high rates of mortality [7,10,11,28].

### 3.2. Management of Odontogenic Necrotizing Fasciitis

Numerous authors have pointed out that early diagnosis and early initiation of adequate therapy are crucial for the outcome of necrotizing fasciitis [2,7,10,13,28]. In most cases, a markedly deranged physiology with abnormal blood parameters is observed [10], which is mainly characterized by abnormal elevation of white blood cells and C-reactive protein, impaired blood coagulation and elevated renal parameters [10]. In this context, the LRINEC score was proposed as a risk indicator with a cutoff value of 6 [29], which was also reached or exceeded by our patients in all cases. Although the sensitivity and specificity of this score, based on simple laboratory parameters, are controversially discussed by some authors [10,30], the score may well be used to assess the severity of disease and thus to raise awareness regarding a possibly necrotizing course [10]. Thus, in our study, the patient that we could not cure had an LRINEC score of 13 on admission. A prompt CT diagnosis of the head and neck region and the thorax with a contrast medium can show the extent of the inflammation and usually allow a reliable differentiation from the common odontogenic abscess [31]. Surgical therapy should be performed as soon as possible, but immediately as emergency therapy in the presence of purplish spots, dark hemorrhagic blisters or definite skin necrosis. In contrast to an odontogenic abscess, necrotizing fasciitis shows significantly reduced tissue resistance, allowing the blunt finger to be advanced beneath the already affected skin without any resistance. Here, it is of utmost importance to expose the affected fascia and to completely remove already necrotized tissue. It is advisable to collect several samples (swabs and native tissue) for microbiological and histopathological examination. Since the detection of anaerobes is often challenging, the microbiologist should be informed in advance and care should be taken to ensure rapid and proper sample transport to the microbiological laboratory [16]. Sampling should ideally take place prior to the first application of antibiotic therapy. Wounds should be treated with negative wound pressure therapy to ensure continuous removal of bacterial enzymes and metabolites from the affected tissues. Postoperatively, patients should receive intensive medical care, and at least one second-look surgical intervention should be performed on the next day to safely remove any further developing necrosis [7]. In our experience, more extensive cases require several interventions until the development of new necrosis is arrested. With regard to the recently observed increase in antibiotic resistance in odontogenic abscesses [16,32], a broad-spectrum antibiotic therapy, for example, with piperamcillin/tazobacam or meropenem should initially be performed. In cases of a pre-existing colonization of the oral cavity with a methicillin-resistant *Staphylococcus aureus* (*MRSA*), additional administration of vancomycin may be considered. In this way, it can be assumed that the entire bacterial community of the infection is safely targeted. If the microbiological findings including an antibiogram are available after several hours, therapy can be adjusted specifically. As recommended by Tsitsilonis et al. [21], clindamycin may be used in addition to the broad-spectrum antibiotic to inhibit bacterial protein biosynthesis in order to retard bacterial production of destructive enzymes. Tracheostomy may be necessary as part of intensive care therapy. However, it is advisable to perform this only if the approach to the trachea is clean and free of necrosis to prevent possible seeding of the bacteria into the mediastinum, which would lead to a very poor prognosis [8]. If no further necrosis becomes apparent after several surgical interventions over several days, therapy with meshed skin grafts can be performed similarly to burn patients. Minor defects confined to the neck can be left to free granulation.

### 3.3. Microbiology of Odontogenic Necrotizing Fasciitis

Odontogenic infections are regularly caused by the bacteria of the oral microbiome [15]. In contrast to inoculation of bacteria through the skin [33], these are endogenous infections. Numerous authors have pointed out that odontogenic infections are caused polymicrobially by aerobic and anaerobic bacteria. Aderhold et al. already pointed out in 1981, and Eckert et al. in 2000, the particular importance of the anaerobic species *Prevotella*, *Porphyromonas*, *Fusobacterium* and *Peptostreptococcus* [19,34]. Recent studies using molecular methods to determine the microbiome of odontogenic infections have confirmed these findings [14,16,17]. In addition to the previously mentioned bacteria, *Parvimonas* has also been associated with strong pathogenicity [18]. In their review about odontogenic infections, Lewis et al. described that culture-based analyses often only detect viridans-streptococci and staphylococci in odontogenic infections, and that the detection of anaerobic bacteria is only possible using strictly anaerobic and low-contamination techniques [35]. It is therefore not surprising that in a large review of 1235 cases of cervicofacial necrotizing fasciitis, mainly streptococci and staphylococci could be isolated, whereas the detection of anaerobes was markedly less successful [8]. In the routine cultural analyses performed in this study, two out of four cases (patient Nos. 1 and 2) showed the typical picture of an odontogenic infection [16,19,34], consisting of abscess-typical anaerobic species (*Prevotella intermedia* or *Fusobacterium nucleatum*) in combination with alpha-hemolytic streptococci. In patient No. 4, only *Prevotella intermedia* was detected by culture, which, however, can also be considered typical for an odontogenic infection [16,19]. Eckert et al. pointed out that culture is not always able to detect all species involved in an odontogenic infection [34], and the examination of the associated microbiome (Figure 1) of patient No. 4 showed that streptococci were also involved despite the lack of cultural evidence. Overall, three out of four patients showed culture findings that were appropriate and typical for both simple odontogenic infection and odontogenic necrotizing fasciitis [8,19]. In patient No. 3, however, *Actinomyces turicensis* and *Bacteroides thetaiotaomicron* were detected in association with *Staphylococcus epidermidis*. While *Staphylococcus epidermidis* was probably a contamination from the skin [16], *Actinomyces turincensis* has been identified as a bacterium that occurs physiologically in the oral cavity [36], but in rare cases, it can cause severe and sometimes necrotizing inflammations [37,38]. *Bacteroides thetaiotaomicron* is a dominant member of the intestinal microbiota, which is known for its strong adaptability to external conditions and its ability to metabolize various complex polysaccharides [39]. Its ability to strongly increase virulence in polymicrobial infections is also well described [40]. To our knowledge, no case of cervicofacial necrotizing fasciitis with such a bacterial constellation has been described thus far. Therefore, this is the first description of necrotizing fasciitis caused by *Actinomyces turicensis* and *Bacteroides thetaiotaomicron*. However, the additionally prepared microbiome showed a composition of mainly *Porphyromonas*, *Pyramidobacter*, *Peptostreptococcus*, *Parvimonas*, *Veillonella* and *Moryella,* in which *Bacteroides* and *Actinomyces* appeared only in a smaller abundance. Thus, the typical appearance of a polymicrobial odontogenic infection was finally seen even in this case.

As previously published, the DNA-based determination of microbiomes reveals many more bacteria in odontogenic infections than would have been found in a cultural analysis alone [14,15,17]. This also seems to be the case in odontogenic necrotizing fasciitis, although, to our knowledge, no microbiome of a necrotizing fasciitis has been published yet. The here determined microbiomes show many more bacteria than cultural analysis, and the polymicrobial character of the infection is further emphasized. It has also become evident that, similar to common odontogenic abscesses, it is probably not useful to search for a single culprit bacterium that would be responsible for the necrotizing course [16]. Presumably, a pathological community is always involved, which receives its virulence through the synergy of the abilities of the individual species [41]. In this context, it is remarkable that even bacteria not primarily expected in odontogenic infections, such as *Coriobacterium* and *Erysipelothrix*, can be part of this pathological community (Figure 1, patient Nos. 2 and 4). In the case of patient No. 1, the possibility of determining a microbiome was not yet available at that time, and the identification of DNA from Bacillus licheniformis seems likely to be a case of contamination. However, we assume that a comparable microbiome would also have been collected here. In summary, with regard to the ability of a pathological community to form necrotizing fasciitis, it is probably not the presence of a particular bacterium (e.g., streptococci or staphylococci) that is decisive, but rather the co-occurrence of specific virulence factors with regard to the metabolism of polysaccharides and oxygen and the ability to produce certain destructive enzymes [12,28]. In relation to the management of odontogenic necrotizing fasciitis, it should finally be noted that, similar to odontogenic abscesses, microbiological culture analysis may only detect a part of such a culprit bacterial community [16], which could have a negative impact on susceptibility testing. However, especially in cases that do not respond adequately to therapeutic measures, adequate bacterial detection may be crucial for patients’ outcome. Therefore, culture-based routine microbiological diagnostics should be complemented by culture-independent molecular detection methods in the future.

## 4. Materials and Methods

Patients of this cross-sectional, retrospective study were observed in two other studies on odontogenic abscesses at the Department of Oral and Maxillofacial Surgery of the University Hospital of the Justus-Liebig-University Giessen between April 2009 and December 2020. The inclusion criterion for this special investigation was the development of extensive skin necrosis due to an odontogenic infection, which was associated with a significantly worse and extended course of disease in all cases. The patients or their authorized relatives gave their written consent to participate, and the entire project was authorized by the Ethics Committee of the Justus-Liebig-University Giessen (Vote 191/16 and Vote 222/14) [15,42]. Clinical therapy was performed independently of the ongoing studies and was carried out initially either with the diagnosis of extensive odontogenic abscess or necrotizing fasciitis, with the diagnosis of abscess corrected to necrotizing fasciitis in the course of disease. For the study, inflammatory parameters were obtained from the first blood sample after hospital admission, and the Laboratory Risk Indicator for Necrotizing Fasciitis (LRINEC) score was calculated as described by Wong et al. [29]. Histopathological examinations were performed following the hospital’s standard with hematoxylin and eosin stain.

Microbiological specimens were collected for both cultural and molecular biology testing during the first surgical intervention from the surgical wounds. For molecular analysis, pure pus was collected in an Eppendorf tube, and swabs (wrapped fiber swabs with gel-based Amies medium) of the pus were taken for culture. The samples were taken in a standardized manner prior to the first administration of antibiotics. However, it could not be ruled out that the patients had already taken antibiotics prior to hospital admission due to the severity of the disease. Culture and antibiograms were performed according to the hospital’s standard, as previously described [16]. Moreover, in addition to the clinical routine, PCR on prokaryote DNA was performed for the patient treated in 2009, and the microbiome of the pus was determined for the three patients treated since 2016 using 16S-rRNA amplicon sequencing and bioinformatics, as previously described [15,43]. For this purpose, specimens were first stored at −80 °C. Subsequently, bacterial DNA was extracted from the initially frozen samples, and the variable area “V4” of the 16S-rRNA genes was amplified by PCR using primers in the conserved flanking areas with adapters. The resulting amplicons of an approximate length of 350 to 370 bps were processed for next-generation sequencing using the Illumina MiSeq System, as described by the vendor (Illumina). After the sequencing of these amplicons, the bacteria-specific sequences were determined and ready for bioinformatic analysis.

Therefore, paired-end sequence reads were joined, and primer sequences were removed, as previously described [44]. Reads with ambiguous base calls or with homopolymers longer than eight nucleotides were removed, and duplicates were merged and aligned against the SILVA bases’ bacterial reference alignment [45]. Applying the Mothur implementation of the uchime algorithm, chimeric reads were removed, taxonomy was assigned and non-bacterial reads were removed from the analysis. Operational taxonomic units (OTUs) were generated, and taxonomy was reassigned using Mothur. In preparation for the analysis, an OTU table in biom format was created.

The statistical analysis was carried out using Microsoft Excel.

## 5. Conclusions

1. Cervicofacial necrotizing fasciitis is rare compared to common odontogenic abscesses, but in most cases, it is also caused by an odontogenic focus.
2. Early diagnosis and immediate initiation of adequate therapy are crucial for the outcome of odontogenic cervicofacial necrotizing fasciitis.
3. Radical surgical therapy in combination with broad-spectrum antibiotic therapy represents the cornerstone of therapy. Negative wound pressure therapy supports wound purification and may facilitate subsequent coverage with skin grafts.
4. Similar to odontogenic abscesses, odontogenic necrotizing fasciitis represents an endogenous, polymicrobial infection in which anaerobic bacteria of the oral microbiome predominate. A single “culprit bacterium” that triggers the necrotizing course could not be identified even by using molecular pathogen diagnostics.
5. In odontogenic necrotizing fasciitis, molecular pathogen diagnostics were able to detect significantly more bacteria than cultural analysis alone. Molecular methods are predestined to become the gold standard in medical microbiology diagnostics, particularly for polymicrobial infections with a predominance of anaerobic bacteria.

## Figures and Tables

**Table 1:** Clinical course and lab values on admission.

	PAT. NO. 1	PAT. NO. 2	PAT. NO. 3	PAT. NO. 4
**AGE/SEX**	71/F	65/F	74/M	38/M
**ORIGIN**	Empty alveolus of extracted tooth 37	Periodontitis apicalis of the lower incisors	Impacted decayed tooth 48 with apical periodontitis	Impacted decayed tooth 38, residual tooth root 36
**REGION**	Left neck and thorax	Submental, bilateral neck, bilateral thorax, shoulders and axillae	Left neck and thorax	Left submandibular and submental region
**ASSOCIATED DISEASE**	None	None	None	Diabetes mellitus
**CLINICAL SIGNS**	Pain, touch sensitivity, swelling, induration, lockjaw, dysphagia, livid erythema	Black blisters, livid erythema, Somnolenz, Sopor, reduced general condition	Black blisters, anesthesia of the skin, livid erythema, reduced general condition	Pain, touch sensitivity, erythema, swelling, induration, lockjaw, dysphagia
**LEUC**	19.1	40.6	16.1	10.9
**CRP**	245.1	368.61	294.61	472.53
**LRINEC SCORE**	8	13	6	7
**PATHOLOGY**	florid granulating, partly purulent inflammation with tissue meltdown	phlegmonous purulent, hemorrhagic and necrotizing inflammation	Necrotizing, acute phlegmonous purulent inflammation	No tissue sample
**RADIOLOGY**	Air accumulations in subcutaneous and submandibular space	Diffuse swelling, extensive fluid and gas accumulation in the soft tissues of the neck	Diffuse air accumulations in the subcutaneous space	Post-incision, diffuse air accumulation in the subcutaneous space of cheek and neck
**CULTURE**	*Prevotella intermedia*, *Alpha-hemolytic Streptococci*, *Candida albicans*	*Streptococcus anginosus,* *Fusobacterium nucleatum*	*Actinomyces turicensis,* *Bacteroides thetaiotaomicron,* *Staphylococcus epidermidis*	*Prevotella intermedia*
**ANTIBIOTIC RESISTANCE**	*P. intermedia:* *Penicillin, Ampicillin*	*S. anginosus:* *Gentamycin*	*A. turicensis: Levofloxacin, Ciprofloxacin* *S. epiderm.: Tetracyclin*	*P. intermedia:* *Vancomycin*
**LENGHT OF STAY**	25	22	36	24
**ICU**	1	21	20	0
**SURG. INT.**	4	9	5	2
**SEC. SURG.**	Skin graft	Tooth removal Skin graft	Tooth removal Skin graft Tracheostomy	Tooth removal

Pat. No.: patient number; LEUC: leucocytes (giga/l); CRP: C-reactive protein (mg/l); ICU: length of stay in the intensive care unit; SURG. INT.: number of surgical interventions; SEC. SURG.: secondary surgical interventions; Pat. No. 2 died.

## Data Availability

The datasets generated and/or analyzed during the current study are available from the corresponding author upon reasonable request.

## Supplementary Materials

The following supporting information can be downloaded at: https://www.mdpi.com/article/10.3390/pathogens11010078/s1, Table S1: Relative abundances of the bacterial genera in the microbiome of Necrotizing Fasciitis.
