# Supplementary material for: Odontogenic Cervicofacial Necrotizing Fasciitis: Microbiological Characterization and Management of Four Clinical Cases
Source: Pathogens. 2022 Jan 9;11(1):78. doi: 10.3390/pathogens11010078 (PMC8778522; doi:10.3390/pathogens11010078)
Supplement: Supplementary file 1 [file pathogens-11-00078-s001.zip › pathogens-1494762-supplementary.pdf]

pathogens

MDPI

Supplementary Material

Table S1. Relative abundances of the bacterial genera in the microbiome of Necrotizing Fasciitis.

Genus.	Pat. No. 2	Pat. No. 3	Pat. No. 4	Mean
Prevotella	11.5017	6.4085	46.7153	21.5418
Porphyromonas	16.4081	25.7687	0.0092	14.0620
Peptostreptococcus	14.8501	6.3073	8.1825	9.7800
Parvimonas	10.1047	2.3329	3.0086	5.1487
Erysipelothrix	13.9398	0.0000	0.5520	4.8306
Coriobacterium	0.8435	0.0000	12.8167	4.5534
Pyramidobacter	0.0009	13.5196	0.0000	4.5068
Veillonella	1.1946	4.0980	7.9985	4.4304
Fusobacterium	5.4298	0.0000	6.5724	4.0007
Moryella	0.0000	9.9162	0.0000	3.3054
Slackia	6.3279	0.2305	1.7175	2.7586
Peptoniphilus	0.0000	6.2173	0.0000	2.0724
Oceanivirga	0.0000	5.9812	0.0000	1.9937
Atopobium	3.7758	1.6190	0.0000	1.7983
Lachnospira	0.8614	0.3485	3.6128	1.6076
Ezakiella	0.0000	4.3679	0.0000	1.4560
Mogibacterium	3.3645	0.0000	0.9569	1.4405
Bacteroides	0.0715	3.9463	0.0061	1.3413
Filifactor	3.2731	0.0000	0.0000	1.0910
Eubacterium	1.2031	0.2136	1.6469	1.0212
Alloprevotella	0.0047	0.0000	2.5915	0.8654
Oribacterium	0.0047	2.4228	0.0000	0.8092
Dialister	0.0038	0.0000	2.2818	0.7619
Streptococcus	0.2052	0.0000	1.1777	0.4610
Treponema	1.2756	0.0000	0.0031	0.4262
Olsenella	0.0038	1.2704	0.0000	0.4247
Family_XIII_ge	1.2228	0.0000	0.0000	0.4076
DNF00809	0.0000	1.1580	0.0000	0.3860
Corynebacterium	1.0694	0.0000	0.0000	0.3565
Rikenella	1.0477	0.0000	0.0000	0.3492
Selenomonas	0.9809	0.0000	0.0215	0.3341
Prevotella_7	0.0000	0.7982	0.0000	0.2661
Solobacterium	0.0000	0.6127	0.0000	0.2042
Actinomyces	0.0028	0.4666	0.0000	0.1565
Catonella	0.4255	0.0000	0.0000	0.1418
Lachnospiraceae FE2018 group	0.0000	0.4104	0.0000	0.1368
Peptococcus	0.3427	0.0675	0.0000	0.1367
Eggerthia	0.0000	0.3092	0.0000	0.1031
Candidatus Saccharimonas	0.0000	0.2811	0.0000	0.0937
Rikenellaceae RC9 gut group	0.0000	0.2530	0.0000	0.0843
Sphaerochaeta	0.0000	0.2080	0.0000	0.0693
Gemella	0.1845	0.0000	0.0061	0.0635
Escherichia-Shigella	0.0000	0.1518	0.0000	0.0506
Finegoldia	0.0000	0.1349	0.0000	0.0450
Neisseria	0.0038	0.0843	0.0092	0.0324
Family XIII AD3011 group	0.0000	0.0787	0.0000	0.0262
Mycoplasma	0.0000	0.0000	0.0644	0.0215
Campylobacter	0.0235	0.0000	0.0000	0.0078
Rothia	0.0000	0.0000	0.0184	0.0061
Anaerococcus	0.0000	0.0169	0.0000	0.0056
Lactobacillus	0.0038	0.0000	0.0123	0.0054

Fretibacterium	0.0038	0.0000	0.0061	0.0033
Staphylococcus	0.0038	0.0000	0.0031	0.0023
Clostridium	0.0066	0.0000	0.0000	0.0022
Moraxella	0.0038	0.0000	0.0000	0.0013
Listeria	0.0038	0.0000	0.0000	0.0013
Ruminococcus	0.0038	0.0000	0.0000	0.0013
Acidibacter	0.0038	0.0000	0.0000	0.0013
Bifidobacterium	0.0000	0.0000	0.0031	0.0010
Haemophilus	0.0000	0.0000	0.0031	0.0010
Clostridiales bacterium	0.0000	0.0000	0.0031	0.0010
Enterococcus	0.0028	0.0000	0.0000	0.0009
Acinetobacter	0.0019	0.0000	0.0000	0.0006
Pseudomonas	0.0019	0.0000	0.0000	0.0006
Pasteurella	0.0019	0.0000	0.0000	0.0006
Defluviitaleaceae_UCG-012	0.0019	0.0000	0.0000	0.0006
Burkholderia-Paraburkholderia	0.0019	0.0000	0.0000	0.0006
Bradyrhizobium	0.0009	0.0000	0.0000	0.0003
Comamonas	0.0009	0.0000	0.0000	0.0003
Howardella	0.0009	0.0000	0.0000	0.0003
